# Googling Service Boundaries for Endovascular Clot Retrieval (ECR) Hub Hospitals in Metropolitan Sydney

**DOI:** 10.3389/fneur.2019.00708

**Published:** 2019-07-02

**Authors:** Thanh G. Phan, Richard Beare, Velandai Srikanth, Henry Ma

**Affiliations:** ^1^Department of Neurology, Monash Health, Melbourne, VIC, Australia; ^2^Clinical Trials, Imaging and Infomatics (CTI), Division of Stroke and Aging Research Group, Medicine, School of Clinical Sciences, Monash University, Melbourne, VIC, Australia; ^3^Department of Medicine, Frankston Hospital, Peninsula Health, Melbourne, VIC, Australia; ^4^Central Clinical School, Monash University, Melbourne, VIC, Australia; ^5^Developmental Imaging, Murdoch Children Research Institute, Melbourne, VIC, Australia

**Keywords:** clot retrieval, stroke, geospatial, Google Map API, optimization, simulation

## Abstract

**Background and Purpose:** Endovascular clot retrieval (ECR) has revolutionized acute stroke therapy but is expensive to run and staff with accredited interventional neuroradiologists 24/7; consequently, it is only feasible for each metropolitan city to have a minimum number of hubs that is adequate to service the population. This method is applied to search the minimum number of hospitals to be designated as ECR hubs in Sydney as well as the population at risk of stroke reachable within 30 min.

**Methods:** Traveling time from the centroids of each suburbs to five ECR capable hubs [Royal Prince Alfred/RPA, Prince of Wales/POW, Royal North Shore/RNS, Liverpool/LH and Westmead/WH]. This step was performed using *ggmap* package in R to interface with Google Map application program interface (API). Next, we calculate the percentage of suburbs within each catchment in which traveling time to the ECR hub is <30 min. This step was performed for all possible combination of ECR hubs. The maps are available at https://gntem3.shinyapps.io/ambsydney/. The population at risk of stroke was estimated using stroke incident studies in Melbourne and Adelaide.

**Results:** The best 3-hospital combinations are LPH/WH/RNS (82.3, 45.7, and 79.7% of suburbs reachable within 30 min or 187 of 226 suburbs) follow by RPA/LPH/RNS (100.0, 80.9, and 73.1% of suburbs) and LPH/POW/RNS (83.3, 90.7, and 76.6% of suburbs). The best 4-hospital model is LPH/WH/POW/RNS (84.2%, 91.1%, 90.7%, 77.8%). In the 5-hospital model, ECR is available for 191 suburbs within 30 min: LPH (83%), RPA (100%), WH (90.2%), RNS (72.7%), POW (88.9%). Based on 3-hospital model and 15% of patient eligible for ECR, the expected number of cases to be handled by each hospital is 465. This number drops down to 374 if a 4-hospital model is preferred.

**Conclusions:** The simulation studies supported a minimum of 4 ECR hubs servicing Sydney. This model provides data on number of suburbs and population at risk of stroke that can reach these hubs within 30 min.

## Introduction

Recent advances in stroke therapy has generated debate on translating clinical trial findings to service the entire population (1–6). This is an issue which come to the attention of mainstream media from time to time when the ability of the team to provide 24/7 did not meet public expectation (7). This case in 2016 illustrated that even 1 year after publications the ECR trials, the ability to translate findings into clinical practice can remain elusive. It is possible that the enormous logistic required to bring together a highly skilled teams comprising of interventional neuroradiologists (INR), stroke (vascular) neurologists, support staff (anesthetists, neurosurgeons, trainee doctors, radiographers, and nurses), and dedicated angiography suites and bed availability financially supported by the government (8).

In Australia, the state of Victoria and South Australia have set up a statewide service protocol (9) for ECR immediately after the publication of the ECR trials (1–6). This idea is similar to the concept of comprehensive stroke center (CSC) but with a difference that the CSC provide care for the catchment and also outlying rural areas (10). In this framework, two hospitals were designated as ECR hubs from a pool of 4 ECR capable hospitals. These ECR hubs are required to provide a 24 h service not just for patients in their immediate local catchment but also for all residents of the states. Using a data driven approach, we showed that it was possible to service most of Melbourne within 30 min with 2 ECR hubs (11). In this paper, an idealized time of 30 min is used based on the modeling in the redesign of stroke service in London (12). A similar process has been developed in South Australia, with one hospital providing ECR service from a pool of 3 ECR capable hospitals. Such a service is in development in Sydney, New South Wales but as yet an official policy statement has not been released (13). Using method for mapping catchment areas in Melbourne and Adelaide (11), we apply similar approach to search the minimum number of hospitals to be designated as ECR hubs in Sydney. Further, we estimate the ECR case load for each combination of hospital to help determine the minimum number of hospitals required as ECR hubs.

## Methods

### Setting

Sydney is the capital city of the state of New South Wales, Australia with a population density of approximately 407 person per km^2^ in greater Sydney (https://www.abs.gov.au/). Excluding the surrounding parks, the population density is 1237 person per km^2^. The postcodes for metropolitan Sydney are in the range 2000–2234, 2555–2567, 2761–2768. The 2016 census data from each postal area in New South Wales can be obtained from https://datapacks.censusdata.abs.gov.au/datapacks/. The number of strokes was estimated using the census data for each age band and the stroke incidence study in Melbourne and Adelaide (14, 15). This study involves simulations (no patient data are used) and as such received a waiver from the Monash Health Human Research Ethics Committee.

### ECR Capable Hospitals in SYDNEY

**Figure 1:**
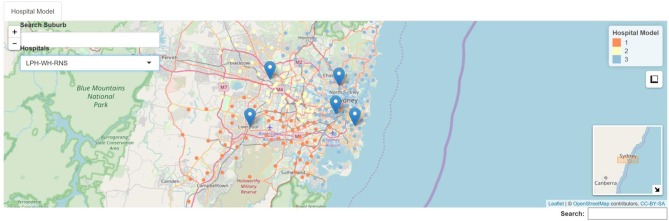
Example of 3 hospital model. RNS, Royal North Shore Hospital, light blue; LPH, Liverpool Hospital, orange; WH, Westmead Hospital, light yellow.

**Figure 2:**
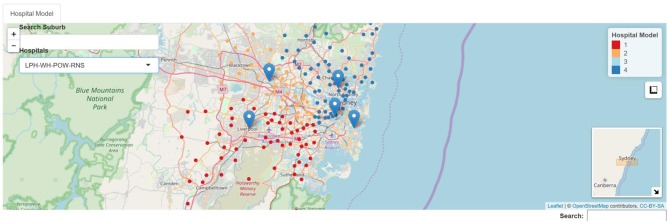
Example of 4 hospital model. RNS, Royal North Shore Hospital, blue; LPH, Liverpool Hospital, red; WH, Westmead Hospital, orange, POW, Prince of Wales Hospital, light blue.

At present there is no official statewide protocol for ECR in Sydney (https://www.aci.health.nsw.gov.au/networks/stroke). There are five hospitals (Royal Prince Alfred/RPA, Prince of Wales/POW, Royal North Shore/RNS, Liverpool/LH and Westmead/WH) capable of acting as ECR hubs. The John Hunter Hospital provides ECR service in Newcastle, 162 km to the North of Sydney. This hospital (postcode 2305) is not considered as part of Sydney catchment. Each of the ECR capable hospitals were considered as a potential ECR hub for the simulation. Membership of a hub was determined using logical comparison of traveling time from each address to the different ECR hospitals. Within each catchment, we calculate the percentage of suburbs in which traveling time to the ECR hub is <30 min. This step was performed for all possible combinations of ECR hubs. The locations of the ECR capable hospitals relative to these arterial roads can be seen in Figures 1, 2. The best combination of ECR hubs were defined according to travel time.

### Google Map API

We used *ggmap* package R (R Project for Statistical Computing, version 3.4.4) to query Google Map application program interface (API) (https://developers.google.com/maps/) (16). The transport times to each hospital from each suburb centroid were computed for morning peak hour traffic.

### *Shiny* and *Leaflet* Display

Results of analyses were displayed as interactive web-based maps using R package leaflet and tiles from OpenStreetMap (^©^OpenStreetMap contributors. For copyright see www.openstreetmap.org/copyright) (17). These maps were uploaded using *Shiny* onto the web (RStudio Inc) available at https://gntem3.shinyapps.io/ambsydney/.

## Results

The results are available in Table 1 and on the web. The best 3-hospital combinations are LPH/WH/RNS (82.3, 45.7, and 79.7% of suburbs reachable within 30 min or 187 of 226 suburbs) follow by RPA/LPH/RNS (100.0, 80.9, and 73.1% of suburbs reachable within 30 min, 184 of 226 suburbs) and LPH/POW/RNS (83.3, 90.7, and 76.6% of suburbs reachable within 30 min, 184 of 226 suburbs).

**Table 1:** Traveling time and coverage area for different combinations of ECR hub models in metropolitan Sydney.

	**Percentage of suburbs within 30 min of RPA**	**RPA suburbs**	**Percentage of suburbs within 30 min of LPH**	**LPH suburbs**	**Percentage of suburbs within 30 min of WH**	**WH suburbs**	**Percentage of suburbs within 30 min of POW**	**POW suburbs**	**Percentage of suburbs within 30 min of RNS**	**RNS suburbs**	**Total number of suburbs serviced within 30 min**	Total number of patients serviced within 30 min^*^	**Total number of patients serviced within 30 min^#^**
3 hospitals-1	76.1	109	77.6	58	76.3	59					173	8559	4997
3 hospitals-2	57.8	109	71.1	76			87.8	41			153	7118	4156
3 hospitals-3	100	50	80.9	68					73.1	108	184	8979	5227
3 hospitals-4	68	75			66.3	92	64.4	59			150	7390	4313
3 hospitals-5	75.9	54					45.3	75	63.9	97	137	6322	3678
3 hospitals-6			83.3	72			90.7	43	76.6	111	184	8982	5228
3 hospitals-7			84.2	57	39.1	69	24	100			174	8624	5034
3 hospitals-8			82.3	62	45.7	46			79.7	118	187^*^	9292	5412
3 hospitals-9					71.2	80	33.8	65	77.8	81	164	8092	4713
4 hospitals-1	67.6	74	83	53	76.3	59	90	40			175	8641	5044
4 hospitals-2	100	42	81.8	55	90.2	41			73.9	88	189	9363	5456
4 hospitals-3	100	38	81.8	66			88.9	36	72.1	86	186	9061	5274
4 hospitals-4	100	31			71.8	74	61.1	55	72.7	66	166	8193	4772
4 hospitals-5			84.2	57	91.1	45	90.7	43	77.8	81	200^*^	9991	5815
5 hospitals-1	100	30	83	53	90.2	41	88.9	36	72.7	66	191	7023	4106

* Melbourne stroke incident study;

# *Adelaide stroke incident study*.

The best 4-hospital model is LPH/WH/POW/RNS (84.2%, 91.1%, 90.7%, 77.8% reachable within 30 min or 200 of 226 suburbs). This model provides better coverage than the next best 4-hospital model (RPA/LPH/WH/RNS) by 11 suburbs and the next best 3-hospital model (LPH/WH/RNS) by 13 suburbs.

In the 5-hospital model, ECR is available for 191 suburbs within 30 min: LPH (83%), RPA (100%), WH (90.2%), RNS (72.7%), POW (88.9%). The 5-hospital model is not superior to the 4-hospital model because RPA is situated in the center of LPH/WH/POW/RNS.

The estimated number of patients that can be treated with each model for each hospital are provided in Table 2. The maximum estimated population reachable within 30 min is observed in 3-hospital model 8 (LPH/WH/POW−9292 cases), follow by model 6 (LPH/POW/RNS−8982 cases) and by model 1 (RPA/LPH/WH−8559 cases). The maximum estimated population reachable within 30 min is observed in 4-hospital model 5 (LPH/WH/POW/RNS−9991 cases), follow by model 2 (RPA/LPH/WH/RNS−9363 cases).

**Table 2:** Projected number of patients with stroke serviced by different combinations of ECR hubs.

	Stroke number for RPA^*^ using Melbourne incident data	**Stroke number for RPA**^#^** using Adelaide incident data**	**Percentage of suburbs within 30 min of LPH**	**LPH suburbs**	**Percentage of suburbs within 30 min of WH**	**WH suburbs**	**Percentage of suburbs within 30 min of POW**	**POW suburbs**	**Percentage of suburbs within 30 min of RNS**	**RNS suburbs**	**Total number of suburbs serviced within 30 min**	Total number of patients serviced within 30 min min^*^	**Total number of patients serviced within 30 min**^#^****
3 hospitals-1	3439	2013	2559	1487	2561	1498	0	0	0	0	173	8559	4997
3 hospitals-2	2425	1415	3105	1808	0	0	1588	933	0	0	153	7118	4156
3 hospitals-3	1759	1043	3148	1833	0	0	0	0	4073	2352	184	8979	5227
3 hospitals-4	2089	1216	0	0	3581	2088	1721	1010	0	0	150	7390	4313
3 hospitals-5	1452	855	0	0	0	0	1578	921	3293	1902	137	6322	3678
3 hospitals-6	0	0	3364	1957	0	0	1627	955	3991	2316	184	8982	5228
3 hospitals-7	0	0	2733	1587	3035	1769	2856	1678	0	0	174	8624	5034
3 hospitals-8	0	0	2837	1647	2270	1332	0	0	4185	2433	187	9292	5412
3 hospitals-9	0	0	0	0	3254	1900	1987	1161	2851	1652	164	8092	4713
4 hospitals-1	1976	1151	2516	1462	2561	1498	1588	933	0	0	175	8641	5044
4 hospitals-2	1544	914	2559	1487	2058	1208	0	0	3202	1847	189	9363	5456
4 hospitals-3	1218	720	3105	1808	0	0	1446	844	3293	1902	186	9061	5274
4 hospitals-4	1116	656	0	0	3077	1798	1578	921	2422	1398	166	8193	4772
4 hospitals-5	0	0	3279	1899	2234	1310	1627	955	2851	1652	200	9991	5815
5 hospitals-1	1003	591	2516	1462	2058	1208	1446	844	2422	1398	191	9445	5503

* Melbourne stroke incident study;

# *Adelaide stroke incident study. The shaded rows represent the combinations with the best coverage*.

The number are lower if based on the Adelaide stroke incident study (15) and higher if based on Melbourne stroke incident study (see Table 1) (14). These hospitals serviced the largest population at risk of stroke. Based on 3-hospital model and 15% of patient eligible for ECR, the expected number of cases to be handled by each hospital is 465. This number drops down to 374 if a 4-hospital model is preferred.

## Discussion

In this study, we have used data driven method to map service boundaries for ECR hubs based on Google Map API estimate of travel time to the hub in Sydney. We estimated that the minimum number of hubs to service Sydney is 4 based on the number of suburbs and population at risk. Further below we will discuss if it is possible to have 4 ECR hubs given availability of INR in Sydney, Australia. Our extension of the methodology to estimation of population at risk will be useful for planning capacity of health services.

In our initial publication on the map of ECR hub catchment for Melbourne, we were challenged by the reviewers to demonstrate that the method could be applied elsewhere (11). We subsequently added the catchment area of ECR hubs for Adelaide, Australia, and now Sydney, Australia (11). Other investigators have described the use of Google Map API for modeling stroke services in North America (18). As such, we postulate that the method can be applied to any international locations serviced by Google Map API and for any time-dependent conditions such as acute coronary syndrome (19). There are several countries, such as Republic of Korea (South Korea) and People's Republic of China, where Google Map API may not be the optimal tool; Google Map API is available for use in Republic of China (Taiwan).

The use of geographical information systems for health service design is evolving since 2017 with several publications in the journal *Stroke* and *Jama Neurology* (11, 18, 20–22). In this study, we have accessed Google Map API for traffic data due to our familiarity with this platform (11). There are other platforms such as Bing Map API (tarifX and tarifX.geo packages in R), Yahoo (https://github.com/trestletech/rydn) and Baidu Map (https://github.com/badbye/baidumap). Outside of the R environment, there is package in Python for performing geospatial analysis (23) and investigators have also used Matlab (MathWorks^@^) for accessing Google Map API (18). Google Map API has a constraint in that it allows only 2500 queries per day; it charges for additional queries.

The data driven method described here show that selection of ECR hubs for other cities cannot be empirically inferred directly from the Melbourne or Adelaide models. This is likely the case since each city has their individual geography, arterial roads, and locations of ECR capable hospitals. Using the idealistic notion of a maximum 30 min traveling time to the ECR hospital, we can assess the ECR hub location which best suits this requirement. In Sydney, it would appear that the optimal models for 3-hospital and 4-hospital models are combinations of LPH/WH/RNS and LPH/WH/POW/RNS. The suburbs serviced by these hospitals have the largest population at risk of stroke. It is likely that the geography of Sydney makes it difficult to service the population with a small number of ECR hubs such as the case in Melbourne. By comparison, >85% of patients can arrive within 30 min with just 2 ECR hub model in Melbourne (11).

The issue of transport to the most appropriate hospital has been the focus of recent research regarding “drip and ship vs. direct to comprehensive stroke center” (20, 24). These strategies were not evaluated here as that is not the intention of this study. A question arises as to the compatibility of the approach based on 30 min trip to ECR hub and “drip and ship” model of care. Our model provides a mean to address this question directly by showing that a large part of Sydney can be reached within 30 min and thus a direct trip to “mothership” ECR hub is possible. These estimates of the population at risk should not be seen as an endorsement of the strategies of sending all patients to ECR hubs. Rather the estimates are provided as a mean to evaluate the capacity of the system to handle the ECR case load. The ability to handle all caseload is part of the equation in the approach to “drip and ship” vs. direct to “mothership” hub conundrum. There has been an increase number of ECR cases since 2015 (25). However, it has been estimated that approximately 84 to 90% cases do not go on to ECR. In this situation the medical stroke code team can be overwhelmed unless some strategies are put in place to limit the transfer of cases to ECR hub (26). A similar strategy that takes into account the population serviced is used in the evaluation of stroke service in England (27). Even with 4-hospital model the average annual ECR case load is 374. This number is a significant challenge to individual ECR hub and a centralized flexible model is required to return patients to a non-ECR hospital nearest to their residential addresses after acute clot retrieval (12). At present such a flexible model had been described with the London reconfiguration but the durability of the repatriation model is not known. A model for repatriation does not yet exist for ECR in Australia. The Victorian statewide protocol emphasizes repatriation of patients back to referring hospital after ECR. However, it does not provide a mean to enforce the referring hospital to accept the patient once repatriation at present (28). Stroke experts will need to actively engage with local government, chief executives of all hospitals, including ambulance services to design these aspects of statewide ECR services.

In discussion on ambulance transport, one often hypothesizes that ambulances arrive faster at destination when using lights and sirens compares to estimations of travel time by Google Map API. We had compared the travel time between observed (ambulance) and simulated travel time by Google Map API and found minimal difference (approximately 3.5 ± 2.5 min) (11). In Australia, ambulance officers are required to obey traffic light and signs, and required to stop at traffic light, check road condition before crossing road intersections. Google Map API provides direction in accordance with traffic regulations (no traveling in the opposite lane). In some cases, estimation of travel time was much faster by Google Map API. It is possible that when Google Map API provides estimate of best-case scenario, it simulates traffic condition in which all traffic lights are green. This effect may be akin to the emergency vehicle prioritization (EVP) protocol. To our knowledge the EVP system is being trial in Queensland but has not been implemented in Adelaide, Melbourne nor Sydney (29).

Our study has several limitations. The simulations were made under certain assumptions about the interventional neuroradiologists (INR) workforce. In the development of the Victorian ECR model, each site was required to have at least 3 accredited INR (28). The register of accredited INR in the State of New South Wales is available at this web address (http://www.ccinr.org.au/register accessed 23/3/19). With 13 available INR, the number of ECR hubs that can be served would be 4. Taking into account the ECR hub in John Hunter Hospital, there would be just sufficient INR to staff 3 ECR hubs in Sydney. Our proposed model requires that the interventional neuroradiologists are not simultaneously on-call at two ECR hub and that each hub has two angiograph suites. The issue of INR rostering is not a just a theoretical concern with a recent coronial inquest over availability of INR in Adelaide, Australia (30). In the event of such staffing shortage or angiographic suite being used for another reason, the model does not hold true as there is a time cost of transporting the patient and additional cost of transporting the interventional neuroradiologist. In an earlier study of cardiac revascularization service, we had shown the impact staff residential location on providing timely service (19). In that study only 45% of inner Melbourne and 56% of outer Melbourne staff could reach hospital within 30 min. Such considerations were not performed here as it would require access to personal data. Our emphasis on 30 min to hospital can be interpreted as too stringent for other countries or a need for centralization of service. Investigators reported that access to access to intravenous-capable hospitals within 60 min was available to 81% of US population and 56% had access to endovascular-capable hospitals (31). In this analysis, we have not considered ECR experiences, interventional neuroradiologists or infrastructure at a potential hub. Of the 5 potential hubs in Sydney, only RNS and WH had participated in the recent ECR trial (5, 32). While services within each hub can be re-organized with increased funding, it is possible that a bottle neck in the consideration of a site for ECR hub is the availability of interventional neuroradiologists.

Our use of ggmap package and which relies on Google Map API has several drawbacks. Principally, Google Map cannot be used to predict future traveling time. Future scenarios can be performed using purpose built strategic model for each state of Australia (33). Further, the *ggmap* package requires modification to perform trip estimation at different time of day. Some of the newer packages in R such as *googleway* permits specification of time of travel (34). In this study we were reassured in that the results from *ggmap* had differed from real ambulance trips by 3 to 5 min in an earlier study (11).

Another limitation of our approach is the reliance on population based studies in other cities within Australia to estimate the number of stroke patients (14, 15). These studies showed a decline in the number of stroke cases over time and it is not certain if our projections do not overestimate the number of stroke cases and which can impact on configuration of ECR hubs. One approach would be to use a current estimate of the number of stroke cases in Sydney. This data is not available at the time of this analysis.

In summary, we estimated the minimum number of ECR hubs to service Sydney and the optimal combination of sites. The choice of sites and their combinations depends on the government agencies and policy makers.

## Disclosure

TP is on the Advisory Board of Genzyme on Fabry Disease and has received payment for lectures including service on speakers' bureaus for Bayer, Boehringer Ingelheim, Pfizer and Genzyme.

## Data Availability

The datasets generated for this study are available on request to the corresponding author.

## Author Contributions

TP: design, analysis, and writing manuscript. RB, VS, and HM: writing manuscript.

### Conflict of Interest Statement

The authors declare that the research was conducted in the absence of any commercial or financial relationships that could be construed as a potential conflict of interest.
